# Emergency Relief Chain for Natural Disaster Response Based on Government-Enterprise Coordination

**DOI:** 10.3390/ijerph191811255

**Published:** 2022-09-07

**Authors:** Feiyue Wang, Ziling Xie, Zhongwei Pei, Dingli Liu

**Affiliations:** 1 Institute of Disaster Prevention Science and Safety Technology, School of Civil Engineering, Central South University, Changsha 410075, China; 2 School of Resources and Safety Engineering, Central South University, Changsha 410083, China; 3 Department of Engineering Management, Changsha University of Science and Technology, Changsha 410114, China

**Keywords:** emergency relief chain, government-enterprise, process coordination, transition allocation

## Abstract

Public health and effective risk response cannot be promoted without a coordinated emergency process during a natural disaster. One primary problem with the emergency relief chain is the homogeneous layout of rescue organizations and reserves. There is a need for government-enterprise coordination to enhance the systemic resilience and demand orientation. Therefore, a bi-level multi-phase emergency plan model involving procurement, prepositioning and allocation is proposed. The tradeoff of efficiency, economy and fairness is offered through the multi-objective cellular genetic algorithm (MOCGA). The flood emergency in Hunan Province, China is used as a case study. The impact of multi-objective and coordination mechanisms on the relief chain is discussed. The results show that there is a significant boundary condition for the coordinated location strategy of emergency facilities and that further government coordination over the transition phase can generate optimal relief benefits. Demand orientation is addressed by the proposed model and MOCGA, with the realization of the process coordination in multiple reserves, optimal layout, and transition allocation. The emergency relief chain based on government-enterprise coordination that adapts to the evolution of disasters can provide positive actions for integrated precaution and health security.

## 1. Introduction

The outbreak of natural disasters has a major impact on public health, risk environment and social stability around the world, e.g., the cyclone Idai in Zimbabwe in 2019 [1]. Natural disasters have caused more displacement, poverty, and casualties than any violence or conflict events [2]. In major disaster events, coordination among different entities can improve overall effectiveness and has attracted extensive attention from the international community [3,4]. The Sendai Framework is applied to the risk of disasters caused by natural or man-made hazards and related environmental hazards, which recognizes that the responsibility to reduce disaster risk should be shared [5,6,7]. The United Nations Secretariat has established a special department to coordinate the activities of different groups at disaster sites. As pointed out in the construction plan of material reserve system, the overall coordination of the current emergency relief system is poor [8]. This issue becomes more significant when key organizational contributions are made by entities that are not commonly considered as emergency response agencies [9]. In response to the negative effect of natural hazards and additional environmental considerations, there is an urgent need to establish a new coordinated relief chain that combines routine and emergency actions to facilitate flexible activation and hierarchical rescue.

Emergency collaboration and organizational coordination may be difficult, and theory and practice have confirmed that it should be throughout the whole process of emergency management [10]. This is because it involves multiple stakeholders, such as non-government organizations (NGOs), nearby communities, and governments [11,12]. The structured responses in disaster-like situations are required [13,14,15]. Different forms of emergency coordination are discussed on the basis of game theory, such as, government-social cooperation, cross-regional emergency and post-disaster partnership [16,17,18]. In addition, there are many factors of NGOs in multi-entities coordination, such as reserve strategies, trust types and performance assessment [19,20,21]. Therefore, it is necessary to first clarify the processes involved in coordination, the form of coordination and the features possessed by the coordinating parties. Ultimately, a structured and systematic emergency relief chain should be established.

In humanitarian logistics, coordination can be divided into three parts: procurement, warehousing, and transportation [22]. Relief resources are generally classified into two categories: daily consumed commodities and specialized equipment [23,24]. However, partial coordination has limitations that may severely affect the extent and effectiveness of coordination. In the coordination of material acquisition, the recruitment of social resources is studied [25]. Quantity flexibility contracts and quantity discount contracts are proposed to coordinate ordering activities of humanitarian organizations and suppliers [26,27,28]. In most previous studies, the common strategy for procurement is incomplete coordination and the benefit distribution is used as the basis for decisions. Moreover, few studies have focused on the nature of coordination and the differences in the division of labor between the government and the market. Coordination mechanisms that develop with disasters have rarely been studied directly.

One of the key issues in the relief chain is the appropriate facility layout and network design to find the best location for storing and allocating emergency resources. The placement of procurement resources in pre-positioned depots is considered the optimal option for maximizing the effectiveness of the relief chain [29,30]. The most common approach to solve the facility location problem is to develop a model based on discrete coverage [31,32,33]. Typical location models are proposed to determine the spatial distribution of emergency facilities [34,35,36]. Studies have also been conducted from the perspective of human society, such as carbon emissions and urban shelters [37,38,39]. Particularly, the reliability of the system plays a great role in problems [40,41,42]. However, there is limited research on coordination-based location and the coordination mechanism has not been further studied and elaborated. The location goals should not only be coverage, but also include fairness, efficiency, and reliability. The impact of geometric representations of disaster areas is often ignored, which reduces complexity but leads to potential decision errors. There is a research need for in-depth coordination in which the functions and characteristics of multiple entities are differentiated.

In light of the above considerations, this paper concentrates on the emergency relief chain for natural disasters based on government-enterprise coordination, with procurement, prepositioning and allocation. The effectiveness, fairness and economy are ensured by the bi-level multi-phase model and multi-objective cellular genetic algorithm (MOCGA). The layout laws obtained from the upper location model are applied to the resource allocation of government and enterprises. Moreover, the coordination benefit and influence factors are explored through sensitivity analysis. The critical contribution of this work is that a solution for demand orientation is provided, and that the coordination of government and enterprises is fostered. Facility investments for resilience are studied with a balance of efficiency and economy. The emergency concepts of diversified reserves, process coordination and transition allocation are presented and validated by numerical cases.

## 2. Problem Description

When a major disaster strikes, the challenge is to provide sufficient quantities of appropriate emergency resources exactly when and where they are needed [43]. Despite the rapid supply and robust security of government emergency, there are problems such as unpredictable demand, the homogeneous type, and difficulties in resource rotation. Owing to the derivative trend and social impact of natural disasters, it is far from enough to rely on government emergency relief alone. Furthermore, real-time procurement after a disaster can be costly and risky from a logistical and social perspective [44]. Municipalities and local regions can develop the capacity to respond to crises and catastrophes through the public-private partnership [45,46,47].

The process coordination and transition allocation of relief chains between government and enterprises are innovatively proposed. In the pre-disaster preparation phase, procured resources are stored in pre-positioned emergency facilities. In the initial post-disaster response, physical reserves are utilized for relief due to the suddenness of the natural disaster. Enterprises are then gradually put into production and supply for coordinated relief and transition allocation based on the quantity discount contract. The ultimate purpose of emergency disposal is to restore the normal production and living order in affected areas. In the recovery phase, the government gradually withdraws from the emergency and the driver of resource supply is adjusted to demand orientation. Accordingly, the strong enforceability of the government and the flexibility and economy of enterprises are integrated, with the consideration of different capabilities in the rescue level and coverage scope.

**Figure 1:**
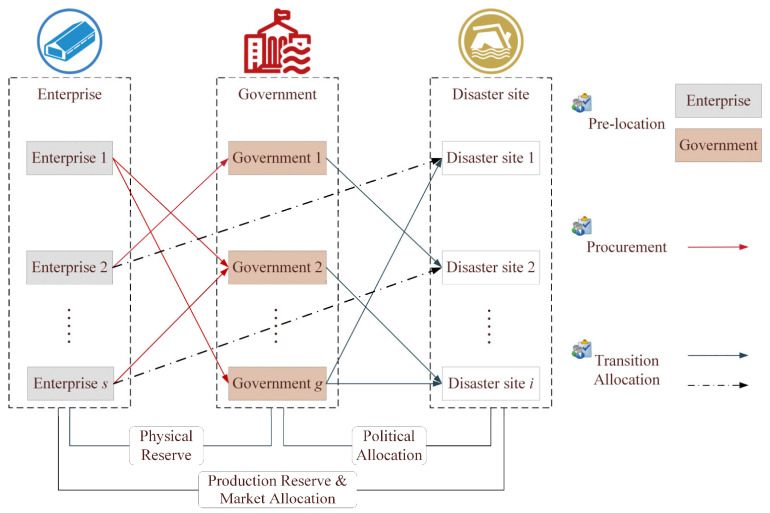
Structure of the emergency relief chain.

The purpose of this study is to develop a reliable layout of emergency facilities based on coordination coverage and transition allocation strategy based on hierarchical emergency for this challenging problem. It is considered as a bi-level multi-phase model of the emergency relief chain based on government-enterprise coordination. Efficiency, fairness, and economy are the goals. Combined with the regional characteristics of disaster sites, a three-echelon emergency relief chain that consists of the government, enterprises and disaster sites has been constructed, as shown in Figure 1.

## 3. Bi-Level Coordination Model of Prepositioning, Procurement and Allocation

### 3.1. Procurement Activities between the Government and Enterprises

There are relatively few studies that address procurement issues in the relief literature, but it is a major challenge for the post-disaster relief chain. In order to reduce the risk of supply disruptions in disasters, it is necessary to establish coordinated inventory management between the government and market through quantity contracts, which could be considered as a form of “virtual stocks” [48].

Consequently, a discount contract for emergency resources is proposed to gain time for production and reduce waste. It allows the government to order a certain quantity of resources and have them pre-positioned in government warehouses, i.e., physical reserves. When a disaster occurs, these resources are transferred through the government to disaster areas. If demand exceeds physical reserves, expedited orders, and produced items are delivered directly from contract enterprises. The complementary role of enterprise production is fully utilized to maximize the supply of resources and establish a benign order.

**Figure 2:**
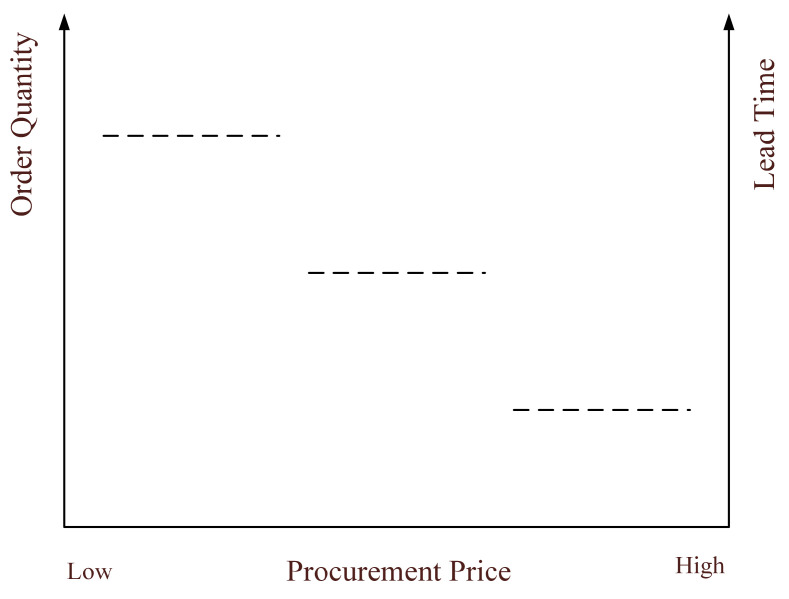
Quantity discount and time discount.

**Figure 3:**
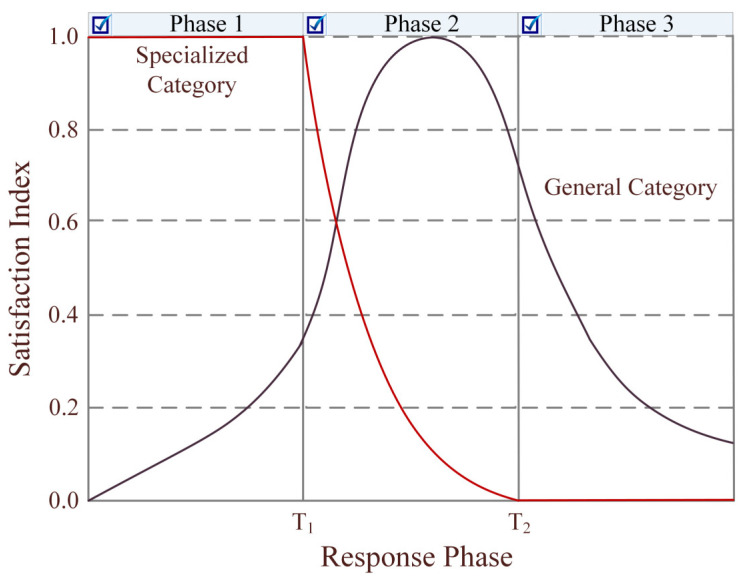
Phased resource benefits.

Discounts are extensively used to coordinate supply chains [49]. It is recommended to combine the quantity-based discount which changes quantity decisions and time-based discount which shifts order time for simultaneous coordination [50]. Accordingly, the procurement price of government is related to the quantity and lead time of the order, as shown in Figure 2. As already suggested and demonstrated, both specialized and general relief items are prepositioned at the government warehouses, while enterprises have the production capability of general items [51]. Disaster phases should be defined by their characteristics rather than time intervals. In particular, the structural damage phase is relatively brief; functional damage refers to the period during which functions are compromised or a loss of functions occurs; recovery is the process of returning system functions to pre-event levels [52]. According to the characteristics of natural disasters and supply-demand relationship, the emergency relief associated with specialized and general categories of resources is divided into three phases, as shown in Figure 3.

### 3.2. Multi-Objective Location Model under Coordination Coverage

**Figure 4:**
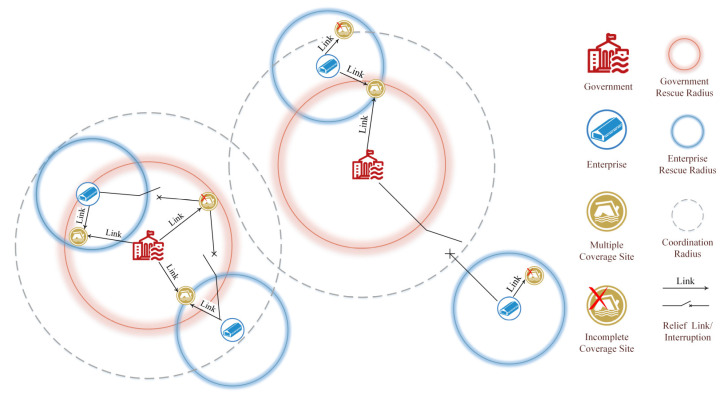
Coordination location of government warehouses and contract enterprises.

The total operation cost of the government emergency is somewhat high because of fixed costs and routine maintenance costs. Since enterprise production is self-operated, the government does not need to input additional construction costs and labor costs. Hence, the coordination between government and enterprises is considered in the actual rescue layout, along with strong execution, flexible supply, and reliable coverage. The linked rescue and coordinated coverage enable isolated relief stations to be connected. Coordination mechanisms are moved forward to the pre-disaster phase, and the location of emergency facilities is studied to provide a theoretical basis for the location-allocation planning problem. The coordination of multiple coverage between government warehouses and contract enterprises is shown in Figure 4.

The main assumptions of this section, which serve as the basis of the proposed model, are as follows:

1. The decision between emergency rescue stations and potential disaster sites is based on the principle of “multiple rescue stations to multiple disaster sites”;
2. Only the coordination relationship between government warehouses and contract enterprises is considered, not the coordination within government warehouses and within enterprises;
3. The weight of the disaster site is considered as follows:

(1) $$w_{i}=\underset{H}{\sum }\alpha_{i}\beta_{i,h}Index_{i,h}\gamma_{i,h}$$

where $\alpha_{i}$ is the population of disaster site i, $\beta_{i,h}$ is the occurrence probability of disaster type h at disaster site i, $Index_{i,h}$ is the demand level of emergency relief for disaster type h at disaster site i, $\gamma_{i,h}$ is the assessment of damage degree for disaster type h at disaster site i;

4. Within the scope of coordination, disaster sites that are covered by government warehouses and contract enterprises with rescue capabilities are considered to be effectively covered.

Furthermore, the following notations are used to formulate the defined problem mathematically:

- Sets.
  G: Set of government warehouses, indexed by g
  S: Set of contract enterprises, indexed by s
  I: Set of disaster sites, indexed by i
  H: Set of emergency disaster type, indexed by h
- Parameters.
  $d_{s,g}$: Transportation distance from contract enterprise s to government warehouse g
  $d_{g,i}$: Transportation distance from government warehouse g to disaster site i
  $d_{s,i}$: Transportation distance from contract enterprise s to disaster site i
  $Q_{i,h}:\;$The occurrence of potential disaster type h at disaster site i
  $C_{g,h}:\;$The rescue capacity of government warehouse g for disaster type h
  $C_{s,h}:\;$The rescue capacity of contract enterprise s for disaster type h
  $R_{g,h}:\;$The rescue radius of government warehouse g for potential disaster type h
  $R_{s,h}:\;$The rescue radius of contract enterprise s for potential disaster type h
  $P_{g,h}:\;$The coordinated rescue scope between government warehouse g and contract enterprise s for the potential disaster type h
  $Area_{i}$: Area of disaster site i
  $Area_{i}^{u}$: Covered area of disaster site i by rescue stations
  $u_{i,h}:\;$Rescue situation for potential disaster type h at disaster site i
  $o_{g,i,h}:\;$The potential disaster type h at disaster site 𝑖 is supported by government warehouse g
  $o_{s,i,h}:\;$The potential disaster type h at disaster site 𝑖 is supported by contract enterprise s
- Variables.
  $y_{g}:$ 1, if government warehouse g is activated; otherwise, 0
  $y_{s}:$ 1, if enterprise s is contracted and put into emergency; otherwise, 0

A multi-objective location model for emergency facilities is proposed to improve the regional emergency response for natural disasters. The objectives are to maximize the coordinated coverage, rescue efficiency, fairness, and economy. Then the impact of objective functions on the location scheme is studied. In addition, rescue level and linkage scope are considered to enhance the applicability and reliability of the emergency location model.

(2) $$f_{1}=\min\overset{I}{\underset{i=1}{\sum }}\overset{H}{\underset{h=1}{\sum }}\frac{Q_{i,h}\left(Area_{i}-Area_{i}^{u}\right)}{Q_{i,h}Area_{i}}$$

(3) $$f_{2}=\min\overset{I}{\underset{i=1}{\sum }}\overset{H}{\underset{h=1}{\sum }}\frac{\sum_{G}o_{g,i,h}d_{g,i}+\sum_{S}o_{s,i,h}d_{s,i}}{u_{i,h}}$$

(4) $$f_{3}=\min\overset{H}{\underset{h=1}{\sum }}\max\left(\max\left(o_{g,i,h}d_{g,i}\right),\max\left(o_{s,i,h}d_{s,i}\right)\right)$$

(5) $$f_{4}=\min\left(\overset{G}{\underset{g=1}{\sum }}y_{g}+\overset{s}{\underset{s=1}{\sum }}y_{s}\right)$$

Subject to

(6) $$y_{g}\in \left\{0,1\right\}$$

(7) $$y_{s}\in \left\{0,1\right\}$$

(8) $$C_{g,h}^{\prime }=\left\{\begin{array}{c} \begin{array}{cc} 1 & \left(C_{g,h}=1\right)and\left(y_{g}=1\right) \end{array}\\ \begin{array}{cc} 0 & \left(C_{g,h}=0\right)or\left(y_{g}=0\right) \end{array}\;\;\; \end{array}\right.$$

(9) $$C_{s,h}^{\prime }=\left\{\begin{array}{c} \begin{array}{cc} 1 & \left(C_{s,h}=1\right)and\left(y_{s}=1\right)and \end{array}\left(d_{g,s}\leq P_{g,h}\right)\\ \begin{array}{cc} 0 & \left(C_{s,h}=0\right)or\left(y_{s}=0\right) \end{array}or\left(d_{g,s}>P_{g,h}\right)\;\;\;\;\;\;\; \end{array}\right.$$

(10) $$o_{g,i,h}=\left\{\begin{array}{c} \begin{array}{cc} 1 & C_{g,h}^{\prime }Q_{i,h}R_{g,h}\geq d_{g,i} \end{array}\\ \begin{array}{cc} 0 & C_{g,h}^{\prime }Q_{i,h}R_{g,h}<d_{g,i} \end{array} \end{array}\right.$$

(11) $$o_{s,i,h}=\left\{\begin{array}{c} \begin{array}{cc} 1 & C_{s,h}^{\prime }Q_{i,h}R_{s,h}\geq d_{s,i} \end{array}\\ \begin{array}{cc} 0 & C_{s,h}^{\prime }Q_{i,h}R_{s,h}<d_{s,i} \end{array} \end{array}\right.$$

(12) $$u_{i,h}=\left\{\begin{array}{c} \begin{array}{cc} 1 & \left(\sum_{G}o_{g,i,h}\geq 1\right)and\left(\sum_{S}o_{s,i,h}\geq 1\right) \end{array}\\ \begin{array}{cc} 0 & \left(\sum_{G}o_{g,i,h}<1\right)or\left(\sum_{S}o_{s,i,h}<1\right) \end{array}\;\; \end{array}\right.$$

Equation (2) is the objective of coordination coverage, which aims to minimize the proportion of uncovered areas to total potential disaster areas in the location scheme. Equation (3) is the objective of efficiency, which aims to minimize the average distance between covered disaster sites and corresponding rescue stations. Equation (4) is the objective of fairness, which aims to minimize the maximum distance of allocation trips. Equation (5) is the objective of economy, which aims to minimize the number of deployed government warehouses and contract enterprises. Equations (6) and (7) show the type of decision variables. Equation (8) shows that the government warehouse g can conduct rescue operations for potential disaster h only if it has been deployed and has corresponding rescue capability. Equation (9) shows that enterprise s can carry out rescue operations for potential disaster h only if it is contracted within the linked coordination scope and has rescue capability. Equations (10) and (11) indicate that the disaster type h at the potential disaster site i is supported if it is located in the rescue scope and the corresponding rescue station has rescue capability. Equation (12) indicates whether the potential disaster type h at disaster site i is covered in coordination.

The above location model for emergency facilities belongs to the non-deterministic polynomial problem. Multi-objective optimization for coverage, efficiency, fairness, and economy is performed based on potential disaster scenarios.

### 3.3. Phased Allocation Model with Quantity Discount Contract

In response to a major natural disaster, the demand for emergency resources in disaster areas may exceed the physical reserves of the local government, in addition to the fact that derived disasters will constantly cause new demand. Therefore, it is necessary to coordinate the production reserves of enterprises in the region for emergency allocation at appropriate phases. Emergency resources can be divided into specialized categories for engineering rescue and general categories for life assistance according to the focus and urgency of the rescue work. An integrated active–passive supply strategy and the transition allocation of physical reserves and production reserves are proposed and applied. A new framework for quantitative tradeoffs between responsiveness and economy is provided.

**Figure 5:**
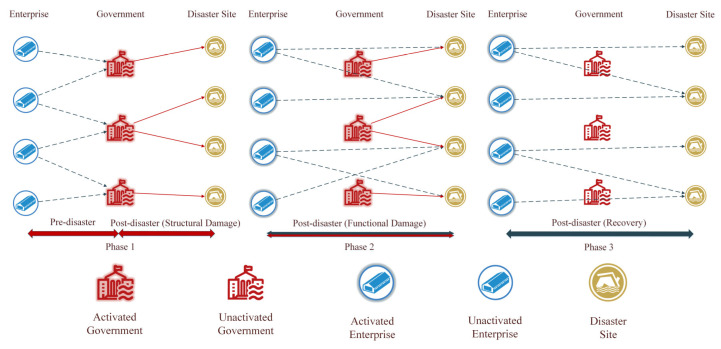
Three-phase resource allocation.

The emergency relief chain consists of three entities: government physical reserves with specialized and general relief capabilities, enterprise production reserves for general items, and disaster sites. The government responds immediately at the moment of the disaster, and enterprises gradually commit in the second phase. After the disaster slowly stabilizes, the government withdraws from the rescue and the supply order is re-established by enterprises. This section defines the phased allocation with the discount contract for government-enterprise coordination, as shown in Figure 5.

The main assumptions of this section, which serve as the basis of the proposed model, are as follows:

1. The layout of government warehouses and contract enterprises is based on the results of the location model;
2. Quantity procurement contracts are negotiated between the government and enterprises to ensure access to supply services before and after disasters;
3. Procurement activities are subject to the price mechanism related to order quantity and lead time;
4. The coordinated supply is composed of reliable enterprises that are not negatively affected by possible disasters in terms of production capacity and emergency response capability;
5. Corresponding penalty costs are incurred due to unmet demand and the residual value of unused inventory is calculated.

Furthermore, the following notations are used to formulate the defined problem mathematically:

- Sets.
  G: Set of government warehouses, indexed by g
  S: Set of contract enterprises, indexed by s
  J: Set of rescue stations containing government warehouses and contract enterprises, indexed by j
  I: Set of disaster sites, indexed by i
  H: Set of emergency resource types, indexed by h
  E: Set of gradients for procurement quantity, indexed by e
  T: Set of phases for post-disaster resource allocation, indexed by t
- Parameters.
  $d_{i,h}^{t}$: Demand for emergency resource h of disaster site i at phase t
  $Q_{g}$: Reserve capacity of government warehouse g
  $M_{s}^{t}$: Production capacity of contract enterprise s at phase t
  $p_{i,m}$: The m-th rescue station that transport emergency resources to disaster site i
  $D_{s,g}$: Transportation distance from contract enterprise s to government warehouse g
  $D_{j,i}$: Transportation distance from rescue station j to disaster site i
  $D_{i}^{max}$: The maximum available distance of disaster site i
  $C_{g}:$ Fixed activation cost for government warehouse g
  $C_{s,h,e}^{t}$: Unit procurement cost of emergency resource h at order level e from contract enterprise s in phase t
  CI: Unit inventory holding cost for government warehouse
  $C_{s,g,h}^{1}$: Unit transportation cost for emergency resource h from contract enterprise s to government warehouse g
  $C_{j,i,h}^{2}:$ Unit transportation cost for emergency resource h from rescue station j to disaster site i
  $C_{i,h}^{3}$: Unit penalty cost of unmet demand for emergency resource h at disaster site i
  $C_{h}^{4}$: Unit salvage value of emergency resource h
- Variables.
  $x_{g}^{t}:$ 1, if government warehouse g is activated at phase t; otherwise, 0
  $y_{s}^{t}$: 1, if contract enterprise s is put into production and relief at phase t; otherwise, 0
  $z_{s,g,h}^{0}$: Distribution quantity of emergency resource h ordered from contract enterprise s to government warehouse g before the disaster
  $z_{j,i,h}^{t}$: Distribution quantity of emergency resource h supplied from relief station j to disaster site i at phase t (containing $k_{s,i,h}^{t}$ and $w_{g,i,h}^{t}$)

Analyzing the demand for emergency resources in typical natural disaster scenarios, the coordination of government physical reserves and enterprise production reserves is proposed. Aiming at rescue efficiency and economy, the allocation strategy of coordinated support, flexible activation and hierarchical response is realized in the process of disaster evolution. The cost components, input phase and coverage scope of emergency entities are different. Eventually, when the disaster is contained at a stable level, government warehouses are gradually shut out and the market order is re-established by enterprises and disaster victims.

(13) $$f_{1}=\min\overset{T}{\underset{t=1}{\sum }}\left(\overset{G}{\underset{g=1}{\sum }}x_{g}^{t}C_{g}+\overset{G}{\underset{g=1}{\sum }}\overset{I}{\underset{i=1}{\sum }}\overset{S}{\underset{s=1}{\sum }}\overset{E}{\underset{e=1}{\sum }}\overset{H}{\underset{h=1}{\sum }}\left(\left(C_{s,h,e}^{0}+CI\right)z_{s,g,h}^{0}+C_{s,h,e}^{t}k_{s,i,h}^{t}\right)\;\;\;\;+\overset{H}{\underset{h=1}{\sum }}(\overset{S}{\underset{s=1}{\sum }}\overset{G}{\underset{g=1}{\sum }}C_{s,g,h}^{1}z_{s,g,h}^{0}+\overset{J}{\underset{j=1}{\sum }}\overset{I}{\underset{i=1}{\sum }}C_{j,i,h}^{2}z_{j,i,h}^{t})\;-\overset{G}{\underset{g=1}{\sum }}\overset{H}{\underset{h=1}{\sum }}C_{h}^{4}\left(\overset{S}{\underset{s=1}{\sum }}z_{s,g,h}^{0}-\overset{I}{\underset{i=1}{\sum }}w_{g,i,h}^{t}\right)\right)$$

(14) $$f_{2}=\min\overset{T}{\underset{t=1}{\sum }}\overset{I}{\underset{i=1}{\sum }}\overset{H}{\underset{h=1}{\sum }}C_{i,h}^{3}\left(u_{i,h,t}^{1}D_{p_{i,1},i}+\overset{J-1}{\underset{m=1}{\sum }}u_{i,h,t}^{m+1}\left(D_{p_{i,m+1},i}-D_{p_{i,m},i}\right)\;+u_{i,h,t}^{J+1}\left(D_{max}-D_{p_{i,J},i}\right)\right)$$

Subject to

(15) $$w_{g,i,h}^{t}\leq z_{s,g,h}^{0}-\overset{T}{\underset{t=1}{\sum }}\overset{I}{\underset{i=1}{\sum }}w_{g,i,h}^{t-1}\leq Q_{g},\;\forall g,h$$

(16) $$k_{s,i,h}^{t}\leq M_{s}^{t}-\overset{T}{\underset{t=1}{\sum }}\overset{I}{\underset{i=1}{\sum }}k_{s,i,h}^{t-1},\forall s,h$$

(17) $$\overset{J}{\underset{j=1}{\sum }}z_{j,i,h}^{t}\leq d_{i,h}^{t},\forall i,h$$

(18) $$u_{i,h,t}^{1}=d_{i,h}^{t*},\;i\in I,\;h\in H,\;t\in T$$

(19) $$u_{i,h,t}^{m+1}=u_{i,h,t}^{1}-\overset{m}{\underset{l=1}{\sum }}z_{p_{i.m},i,h}^{t},\;m\in J,\;i\in I,h\in H,\;t\in T$$

(20) $$d_{i,h}^{t*}=d_{i,h}^{t}+u_{i,h,t-1}^{J+1},\;i\in I,h\in H,t\in T$$

(21) $$\overset{G}{\underset{g=1}{\sum }}x_{g}^{t}+\overset{S}{\underset{s=1}{\sum }}y_{s}^{t}=\left\{\begin{array}{c} \left[0,G\right]\;\;\;\;\;\;\;\;\;\;\;\;\;t=1\\ \left[0,G+S\right]\;\;\;\;1<t\\ \left[0,S\right]\;\;\;\;\;\;\;\;\;\;\;\;\;\;t=T \end{array}\right.<T$$

(22) $$\overset{J}{\underset{j=1}{\sum }}z_{j,i,h}^{t}>0,\;i\in I,\;h\in H,t\in T$$

(23) $$z_{j,i,h}^{t},\;k_{s,i,h}^{t},\;w_{g,i,h}^{t}\geq 0$$

(24) $$x_{g}^{t},\;y_{s}^{t}\in \left[0,1\right]$$

Equation (13) represents the goal of minimizing the total cost of emergency allocation, including five components before and after the disaster: facility activation cost, procurement cost, inventory cost, transportation cost and item salvage value. Equation (14) represents the goal of minimizing disaster losses due to rescue delays, which is directly related to the quantity and arrival sequence of resources. Equation (15) ensures that the supply of government warehouses cannot exceed the available resources. Equation (16) ensures that enterprises do not supply more than their surplus capacity. Equation (17) ensures that the allocated quantities do not exceed the demand of disaster sites. Equations (18) and (19) are progressive updates to the unmet demand. Equation (20) is the demand update at each decision phase, i.e., the cumulative supply margin and the newly added demand. Equation (21) is the quantitative characteristics for transition allocation of government warehouses and contract enterprises. Equation (22) indicates that each disaster site is assigned to at least one rescue station. Equation (23) shows the 0–1 constraint for the decision variables. Equation (24) shows the non-negative constraint.

## 4. Multi-Objective Cellular Genetic Algorithm

The proposed bi-level decision model is a multi-objective model with mixed parameters and conflicting optimization. Heuristic algorithms have better performance compared to exact algorithms [53]. In fact, single-objective algorithms often lead to the degradation of other indicators within the solution set. The multi-objective algorithm is based on the idea that the Pareto front is mutually non-dominant to provide simultaneous optimization. The prior introduction of objective weights as in the weighting method is not required, which avoids subjectivity and saves computational space. The optimal coordinated location for emergency facilities and the phased allocation of emergency resources can be provided to decision makers through population iterations.

**Figure 6:**
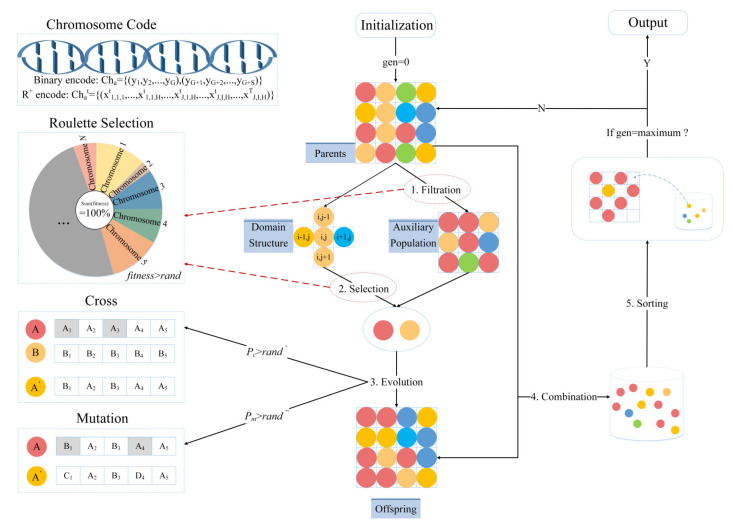
The procedure of MOCGA.

Multi-objective cellular genetic algorithm is applied and adapted, which considers meta-cells as individuals with self-learning ability and evolutionary operations are performed in the domain structure of the cellular automata. In order to avoid the destruction of optimal individuals, the auxiliary population in MOCGA is used to generate offspring populations with the parent population. The algorithm procedure is shown in Figure 6.

### 4.1. Population Code

The decision variables $y_{g},y_{s}$ in emergency facility location are 0–1 variable, indicating whether to deploy government warehouses and contract enterprises in the relief network. Therefore, the binary code is used to better match its actual meaning and each chromosome in the population represents a location scheme of emergency facilities, denoted as $Ch_{a}=\left\{\left(y_{1},y_{2},\ldots ,y_{G}\right),\left(y_{1},y_{2},\ldots ,y_{S}\right)\right\}$.

The decision variables $z_{j,i,h}^{t},\;k_{s,i,h}^{t}$ and $w_{g,i,h}^{t}$ in emergency resource allocation are non-negative real numbers that indicate the transported quantity of emergency resources. For this reason, it is more intuitive to represent the flow of resources during the rescue process with the real number code. Each chromosome in the population represents an allocation scheme of emergency resources, including the supply proportion of government and enterprises in phases, denoted as $ch_{a}^{t}=\left\{z_{1,1,1}^{t},z_{1,1,2}^{t},z_{1,1,H}^{t},\ldots ,z_{J,1,H}^{t},\ldots ,z_{J,I,H}^{t}\ldots ,z_{J,I,H}^{T}\right\}$. An initial population containing NIND chromosomes is then randomly generated.

### 4.2. Fitness Assessment

**Figure 7:**
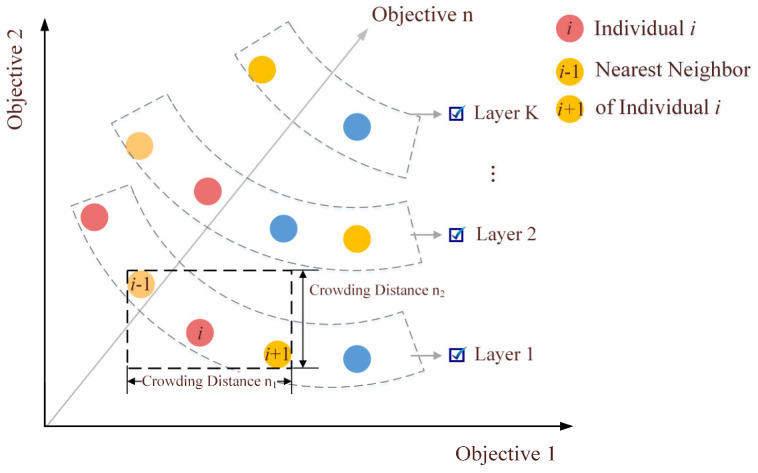
Fitness assessment of non-dominated sorting.

The population scale remains constant during iterations. In order to select the optimal individual in the offspring population, fitness assessment and the ranking operation are required. The fitness evaluation with a single objective is not conducive to global search, so the fast non-dominated ranking in Figure 7 is adopted. Fitness is negatively correlated with the number of layers that individuals are in. Then individuals within the same layer are sorted by crowding distance. The crowding distances of individuals at the two ends of a layer are infinite because they have the maximum or minimum objective values. For the other individuals in the same layer, the fitness value is calculated as:

(25) $$C_{i}=\sum_{n=1}^{N}\frac{f_{n}^{i+1}-f_{n}^{i-1}}{f_{n}^{max}-f_{n}^{min}}$$

where N denotes the number of objective functions, $f_{n}^{i}$ denotes the n-th objective function value of individual i, $f_{n}^{max}$ and $f_{n}^{min}$ denote the maximum and minimum values of the n-th objective function.

### 4.3. Genetic Operation

Genetic operation is a collective term for three types of transformations: “selection” in which strong individuals are selected, “crossover” in which individuals exchange genes to produce new individuals, and “mutation” in which individuals mutate their genetic information. The search process for the optimal solution in the genetic algorithm is conducted by genetic operators.

Individuals are selected from the parent population and the auxiliary population for subsequent evolutionary operations. Roulette selection and neighborhood structure are used to solve the problems of search efficiency and solution accuracy in traditional algorithms. First, the pair of individuals is randomly removed from the pairing pool. Then, one or more integers k in $\left[1,\;L-1\right]$ are randomly selected as the crossover or mutation position according to the string length L. Finally, the operator is implemented by the probability $P_{c}$ or $P_{m}$.

## 5. Numerical Study

### 5.1. Study Area

**Figure 8:**
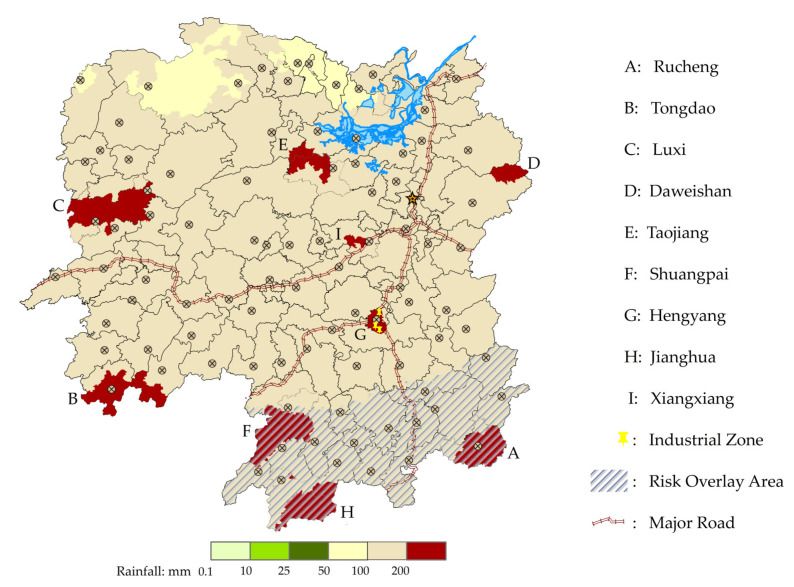
Cases of flood rainfall in Hunan Province, China.

In this section, we demonstrate the applicability of the bi-level model and MOCGA through a series of numerical studies. The high probability of floods and geological disasters are caused by the heavy rainfall in the flood season. Hunan Province is one of the most extensively and severely affected provinces in China, and 18% of its territory belongs to the key flood control areas. The accumulated rainfall in Hunan Province, China, from 22 May to 5 June 2022 is used as the case background. The average rainfall is 170.7 mm, which is 94% more than the normal value (88 mm). Electronic maps supported by geoinformation technology have become a widely used tool for crisis information sharing [54]. Based on the official rainfall data and warning notifications, the distribution of the affected areas is mapped by ArcGIS, as shown in Figure 8. The study area is divided into 97×84 grids, each with 8 km side length.

It is increasingly acknowledged that the extent of losses and damage and the perception of natural disaster risk can be influenced by environment considerations [55]. Supported by the factors affecting disaster emergency response in China, the demand level is determined by the socioeconomic resources, including ethnic cultures and industrial structure [56]. For instance, Xiangtan, with a large number of historic monuments, and Hengyang, with a concentration of industrial zones, are classified as level 1. Besides, the damage degree is determined by environmental conditions, including population density and climatic conditions. In particular, the potential superimposed flash floods and geological hazards are obtained from official data and notifications. The rainfall, population, damage degree and demand level of the disaster areas are shown in Table 1.

### 5.2. Calculation and Analysis of Location

Based on limited relief facilities and restricted relief distances, it is important to improve efficiency, fairness, and coordination. The MOCGA is used to find the optimal scheme for the location model. The population scale is set to 400; the maximum number of iterations is set to 100 and 5 independent simulations are conducted.

#### 5.2.1. Multi-Objective Based Location

The average distance, maximum distance, and coordinated coverage between the rescue stations and potential disaster sites in schemes are shown in Table 2. The overall trend in the average distance reflects precision rescue with the inputs of government warehouses and contract enterprises, ranging from 13.46 to 3.91. The maximum distance objectively increases with the coverage of discrete points, where the minimum value of 9.80 implies uncovered remote sites and the maximum value of 21.73 implies the inefficiency of remote rescue. As observed from the law of complete coverage, government deployment is the prerequisite, and enterprise deployment is the key force. Nevertheless, scheme benefits are not a monotonically increasing function of inputs. For example, the average distance increases by 7.01% when contract enterprises increase from 7 to 8. This reveals that contract enterprises of 7 may be the most economical while securing efficiency. The layout of 4 government warehouses is the threshold to reach stable full coverage.

#### 5.2.2. Coverage Based Location

**Figure 9:**
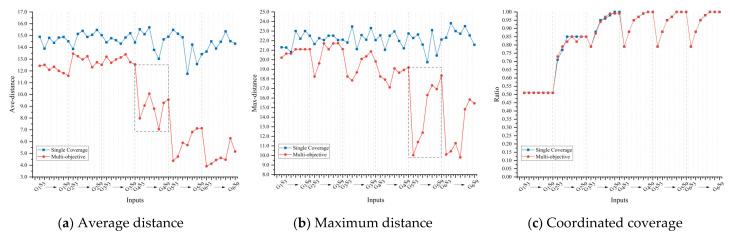
Location strategies between multi-objective and the single coverage.

**Figure 10:**
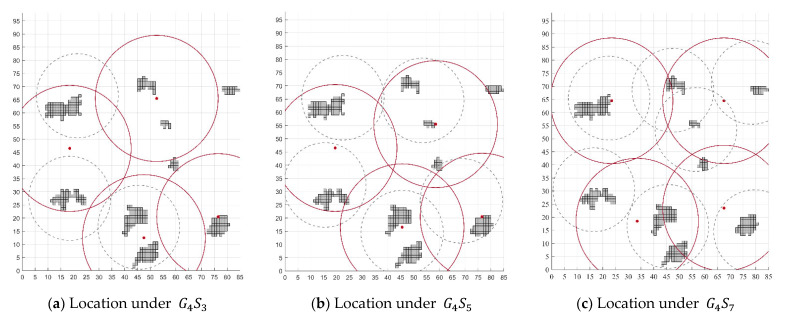
Layout fluctuations in terms of $G_{4}$.

The result comparison of location strategies is illustrated in Figure 9. It can be found that the multi-objective strategy of efficiency, fairness and coordinated coverage considerably outperforms the single coverage strategy. The average distance is reduced by 63.9% and the maximum distance is reduced by 28.3%. Since the simulations are repeated independently, the single strategy is slightly more stable when at the edge of coverage. However, almost the same level of coverage is reached. The change process from $G_{4}S_{3}$ to $G_{4}S_{7}$ is used to illustrate some fluctuations in the values, as shown in Figure 10. There is the “W” fluctuation in distance, such as the varying process of “12.55–7.97–10.07–7.05–9.56” under $G_{4}$. It originates from the inadequate layout of enterprises in the new cycle, as $G_{4}S_{3}$; then increases with rescue operations, as $G_{4}S_{5}$; decreases due to optimal layout, as $G_{4}S_{7}$; and ultimately increases due to redundant coverage. The maximum distance fluctuates dramatically in the later $G_{5}$ phase, indicating that the fairness of remote disaster sites is tricky for inputs.

### 5.3. Calculation and Analysis of Allocation

#### 5.3.1. Allocation in Government-Enterprise Coordination

**Figure 11:**
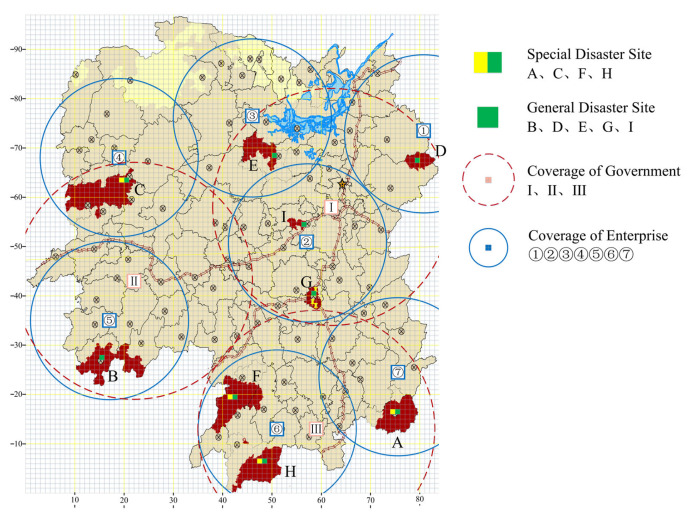
Emergency network design and facility layout.

The layout of emergency facilities with G = 3 and S = 7 is selected as the background of emergency resource allocation, as shown in Figure 11. Allocation schemes play an important role in post-disaster response by allocating scarce resources while minimizing disaster losses and operational costs. The demand of emergency resources generated by the affected population and environment considerations is shown in Table 3, in packaged units. The general resources in shortage after a disaster are produced and supplied by contract enterprises in emergency. The relationships between procurement price and order quantity and lead time are shown in Table 4.

**Figure 12:**
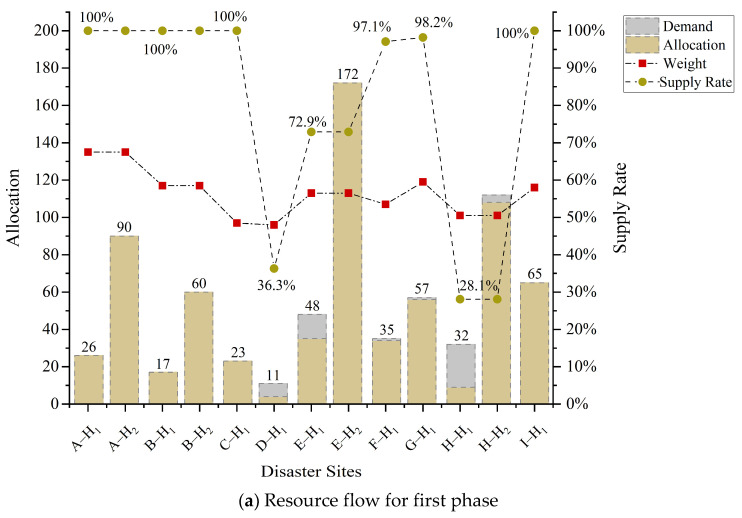

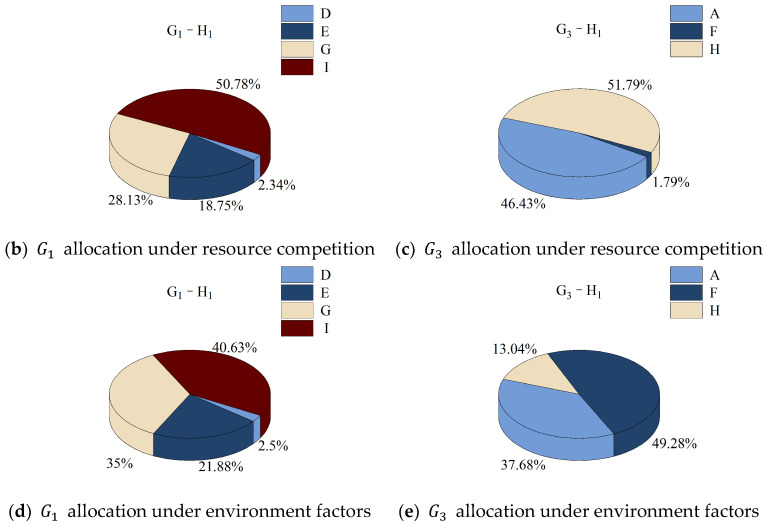
Allocation proportion under the influence of environmental factors.

The resource allocation under the government-enterprise coordination is illustrated in Table 5, and the demand is gradually met through multi-phase decisions and the supply of enterprise production. The shortage of government physical reserves in the first phase is supplemented by enterprises. With the restoration of production capacity and market order, a 100% demand satisfaction rate is eventually achieved. The effect of environmental factors on the allocation results and proportions is shown in Figure 12. It can be seen that in the case of resource scarcity, emergency resources are relatively tilted to the disaster sites with high environmental risks. For instance, the resource supply of sites A, G, and B fulfills 100%, 100% and 98.2%, respectively. The trend of resource satisfaction is consistent with the weight of disaster sites. Under the consideration of environmental factors and game theory, 10.2% of the resources of $G_{1}$ are absorbed to disaster sites E, G, and I, and 47.5% of the resources of $G_{3}$ are absorbed to F.

#### 5.3.2. Strategy Comparison

**Figure 13:**
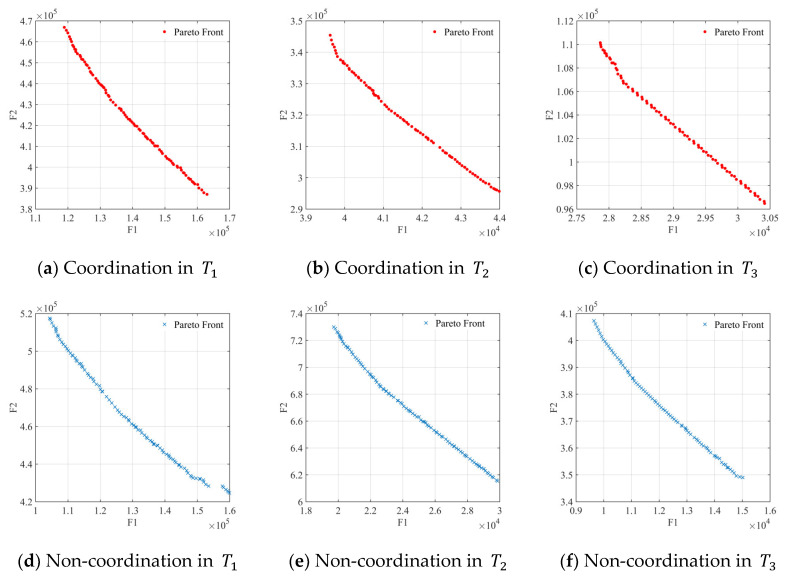
Impact of coordination mechanisms.

In the same disaster scenario, it is assumed that government physical reserves are used for relief without coordination. The premise is to predict the scale of the disaster and reserve adequate emergency resources. In contrast, it can be noted that operation costs and delay losses under the coordination mechanism gradually decrease as the disaster evolves, as shown in Figure 13. At first, relief is carried out by government physical reserves, and the unmet portion of demand can be covered by subsequent production enterprises. Although the demand for resources during the functional damage phase is high, the cost is better controlled and reduced by 25.5% due to the advantages of bulk purchase and no transit. Besides, the delay losses are reduced by 74.1% due to the input of enterprise production. In the post-disaster recovery phase, the government withdraws from rescue, and enterprises and the market carry out normalized supply and slowly transition to the usual state.

The system costs in non-coordination are positively correlated with the trend of disaster derivation and the associated procurement costs are apportioned to each phase. Lower pre-disaster procurement costs do not offset increased warehouse activation costs, inventory costs and transit transportation costs over time, which have increased by 42.3%. The importance of coordination between government physical reserves and enterprise production reserves in relief operations is well captured.

#### 5.3.3. Sensitivity Analysis

**Figure 14:**
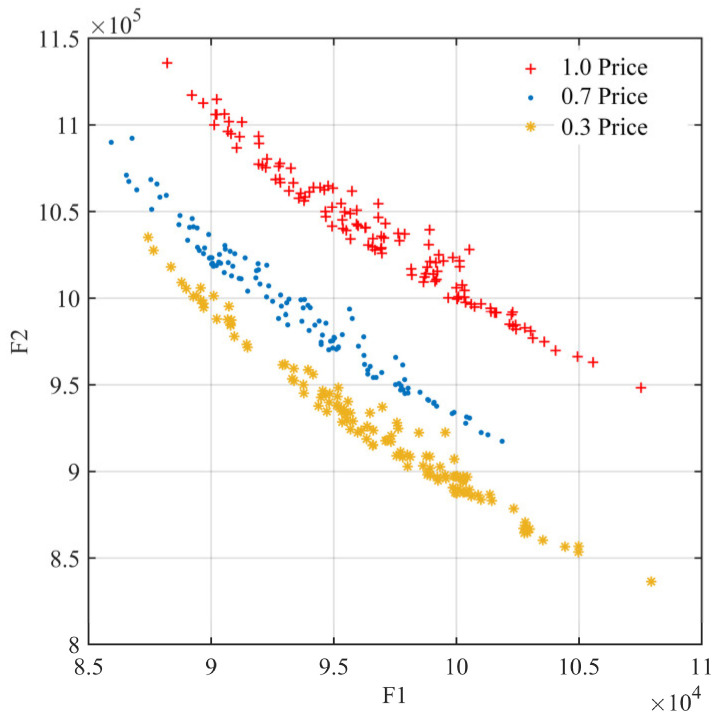
Price factors.

As shown in Figure 14, the initial price reduction for pre-disaster orders has a greater effect on the delay losses and operation costs of emergency relief chain, which are 3.3 and 2.4 times higher than the later reduction. This reflects the large proportion of warehouse operations and cumulative inventory costs in the middle and late phases of disaster relief. It can be considered to reduce pre-disaster procurement prices by bulk sales, improving the target benefits of the relief chain and all entities in the relief chain.

**Figure 15:**
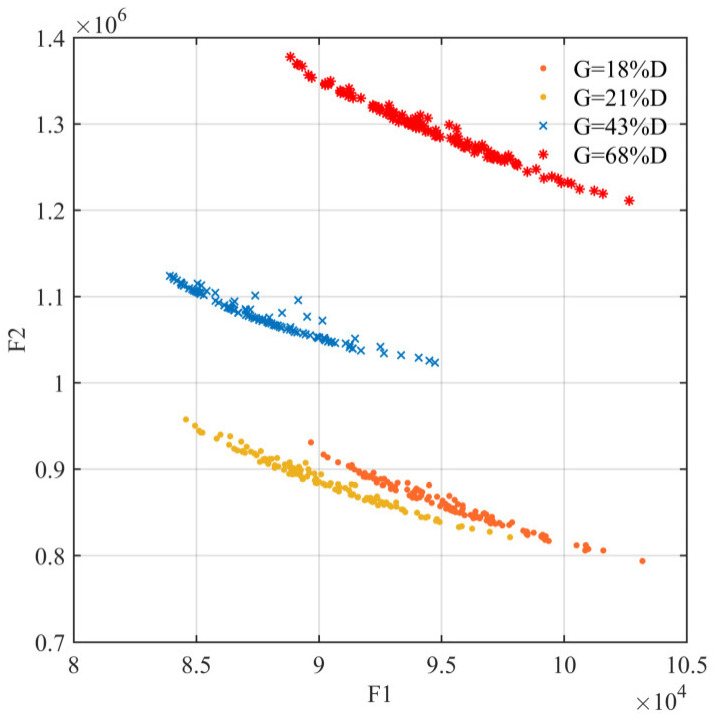
Coordination level.

Government reserves at 18%, 21%, 43% and 68% of total demand reflect the extent of government involvement in the rescue, i.e., partial initial phase, full initial phase, partial transition phase and full transition phase, respectively. From Figure 15 and Table 6, it can be noticed that continuous government coordination to the intermediate transition phase is an optimal choice, which integrates the efficiency of government reserves with the economy of enterprise production. Its allocation scheme has the lowest delay losses and rescue costs, with the composite efficiency index of 611.0. In summary, the continuous and balanced availability of demand-led resources can be delivered by government-enterprise coordination. The supply pressure on government reserves is relieved and the transition to the usual state of the market supply is completed.

### 5.4. Management Insights

Based on numerical studies, some management insights on the emergency relief chain can be extracted as follows:

Synergistic supervision in peacetime and unified command in wartime can be realized by the process coordination of procurement, prepositioning and allocation, which is conducive to optimizing the comprehensive coordination mechanism of the emergency relief chain.

The layout schemes with periodic and regular input benefits can be given by the location strategy based on multiple objectives, and the coverage reliability, rescue efficiency and fairness are ensured.

Demand orientation and disaster control can be covered by the multi-phase transition allocation of resources, where political regulation of government and market mechanism of enterprises are performed. It solves the problem of “reserve composition, dispatch strategy and hierarchical emergency”.

The coordination level is the result of efficiency and economy, where in-depth cooperation and optimal layout can be found for simultaneous optimization. Environmental considerations should be the priority under resource restraints.

There is an essential paradox between the evolution of disasters and the effectiveness of rescue operations. However, the purpose of coordination is to adapt to the occurrence characteristics of disasters and transform them into a decision game.

## 6. Conclusions

The government-enterprise coordination problem in response to natural disasters is presented to avoid the homogeneous layout of rescue organizations and reserves. The key scientific issues of demand orientation, process coordination and transition allocation in the emergency relief chain are implemented. A bi-level multi-phase planning model is proposed, including procurement, prepositioning and allocation. MOCGA is applied to optimize the critical performance indicators of efficiency, economy, and fairness in emergency response. Emergency management capabilities and decision schemes can be acquired by the model and algorithm.

To further explore the operation of the coordination mechanism, numerical simulations are conducted for the flood relief in Hunan Province, China. The multi-objective location strategy is proven to be better, with 63.9% and 28.3% reduction in average and maximum distance, respectively. There are “W” fluctuations in the input benefits of infrastructures and the boundary conditions of reliable coverage are identified. The allocation of emergency resources under government-enterprise coordination can improve the efficiency and economy and relieve the supply pressure. It has been verified that the system performance is optimal when the government coordination is deep into the intermediate transition phase.

The process coordination of government and enterprise is established, which consists of prepositioning, procurement, and allocation. First, the boundary conditions of optimal layout and reliable coverage of emergency facilities are explored by the multi-objective model. Further, the sustainable and balanced availability of demand-oriented resources is conducted through multiple reserves, and the market supply order of recovery phase is re-established. Then, the evolutionary characteristics of disasters and the capacity and nature of emergency entities are fully accounted for, thus completing the multi-phase transition allocation and hierarchical emergency. Ultimately, the resilience of the system for disaster prevention and mitigation is improved. This study makes the following priorities of disaster risk reduction: understand and analyze disaster risk in all its dimensions of vulnerability, capacity, exposure of persons, hazard characteristics and environment; foster collaboration and partnership of government and enterprise for mitigation, preparedness, response and recovery; and research facility investments for resilience, balancing efficiency and economy.

There are some limitations, in that the disruptions of paths and enterprise supply are not considered. In future work, the supply stability and vehicle path planning can be expanded as part of the model. The government-enterprise coordination and transition allocation identified in this paper could provide a basis for the emergency response to the pandemic disaster. Furthermore, the coordination based on other types of supply contracts and logistics in the relief chain can be studied.

## Figures and Tables

**Table 1:** Related data of disaster areas.

Sites	Rainfall/mm	Population/Thousand	Damage Degree	Demand Level	$w_{i}$
A. Rucheng	617.9	360.0	Ⅰ	Ⅲ	(1)
B. Tongdao	566.4	239.8	Ⅲ	Ⅱ	(3)
C. Luxi	450.0	320.0	Ⅲ	Ⅲ	(8)
D. Daweishan	438.5	155.0	Ⅱ	Ⅱ	(9)
E. Taojiang	432.9	68.56	Ⅱ	Ⅲ	(5)
F. Shuangpai	416.6	49.67	Ⅰ	Ⅲ	(6)
G. Hengyang	411.5	79.81	Ⅲ	Ⅰ	(2)
H. Jianghua	367.3	44.82	Ⅰ	Ⅱ	(7)
I. Xiangxiang	343.3	90.96	Ⅱ	Ⅰ	(4)

**Table 2:** Multiple objectives of location schemes.

Item	Government Physical Reserves	Enterprise Production Reserves						
		S = 3	S = 4	S = 5	S = 6	S = 7	S = 8	S = 9
Average Distance (×8 km)	G = 1	12.43	12.51	12.09	12.35	12.00	11.81	11.59
	G = 2	13.46	13.24	12.97	13.24	12.31	12.73	12.52
	G = 3	13.20	12.69	12.97	13.14	13.40	12.74	12.55
	G = 4	7.97	9.06	10.07	8.80	7.05	9.29	9.56
	G = 5	4.38	4.73	5.95	5.70	6.81	7.12	7.13
	G = 6	3.91	4.12	4.44	4.62	4.47	6.28	5.16
Maximum Distance (×8 km)	G = 1	20.22	20.64	20.64	21.10	21.10	21.10	21.10
	G = 2	18.25	19.62	21.71	21.10	21.71	21.73	21.10
	G = 3	18.25	17.84	18.68	20.09	20.35	20.878	19.82
	G = 4	18.25	17.93	17.11	19.09	18.65	18.93	19.20
	G = 5	10.03	11.40	12.38	16.31	17.32	16.94	18.34
	G = 6	10.10	10.44	11.28	9.80	14.82	15.84	15.45

Notes: the location schemes with 100% coverage in the repeated simulations are surrounded by dashed lines.

**Table 3:** The demand of emergency resources in phases.

Disaster Sites	Coordinates	T=1		T=2		T=3	
		H=1	H=2	H=1	H=2	H=1	H=2
$I_{1}$	(76,17)	26	90	60	32	40	0
$I_{2}$	(16,28)	17	60	40	22	27	0
$I_{3}$	(21,64)	23	0	54	0	36	0
$I_{4}$	(80,68)	11	0	26	0	17	0
$I_{5}$	(51,69)	48	172	114	62	76	0
$I_{6}$	(43,20)	35	0	83	0	55	0
$I_{7}$	(59,41)	57	0	133	72	88	0
$I_{8}$	(49,7)	32	112	75	41	49	0
$I_{9}$	(57,55)	65	0	152	0	101	0

**Table 4:** Procurement price mechanism.

Quantity	Pre-Disaster	Emergency	Recovery
0–40	20	35	26
40–100	17	28	21
100–	15	25	16

**Table 5:** Emergency allocation flow under government-enterprise coordination.

Phases		A		B		C		D		E		F		G		H		I	
		$H_{1}$	$H_{2}$	$H_{1}$	$H_{2}$	$H_{1}$	$H_{2}$	$H_{1}$	$H_{2}$	$H_{1}$	$H_{2}$	$H_{1}$	$H_{2}$	$H_{1}$	$H_{2}$	$H_{1}$	$H_{2}$	$H_{1}$	$H_{2}$
Phase 1	G	26	90	17	60	23	0	4	0	35	172	34	0	56	0	9	108	65	0
	S	–	–	–	–	–	–	–	–	–	–	–	–	–	–	–	–	–	–
	Rate	100%		100%		100%		36.4%		94.1%		97.1%		98.2%		81.3%		100%	
Phase 2	G	0	30	0	22	0	0	0	0	0	62	0	0	0	72	0	44	0	0
	S	60	0	40	0	54	0	33	0	127	0	84	0	134	0	98	0	152	0
	Rate	97.8%		100%		100%		100%		100%		100%		100%		99.3%		100%	
Phase 3	G	–	–	–	–	–	–	–	–	–	–	–	–	–	–	–	–	–	–
	S	40	0	27	0	36	0	17	0	76	0	55	0	88	0	49	0	101	0
	Rate	100%		100%		100%		100%		100%		100%		100%		100%		100%	

**Table 6:** Program benefits under the coordination levels.

Coordination Level	Delay Loss (×10^4^)	Rescue Cost (×10^5^)	Economic Index
G = 18%D	8.97	9.31	629.0
G = 21%D	8.46	9.58	629.5
G = 43%D	6.03	9.28	611.0
G = 68%D	8.88	13.8	903.4

## Data Availability

The data presented in this study are available upon request from the corresponding author.
